# Editorial: Comorbidity Burden in Rheumatic Diseases

**DOI:** 10.3389/fmed.2018.00197

**Published:** 2018-07-03

**Authors:** Elena Nikiphorou, Michael T. Nurmohamed, Zoltan Szekanecz

**Affiliations:** ^1^Academic Rheumatology Department, King's College London, London, United Kingdom; ^2^Rheumatology Department, Whittington Hospital, London, United Kingdom; ^3^Medical Center, VU University Amsterdam, Amsterdam, Netherlands; ^4^Division of Rheumatology, Faculty of Medicine, University of Debrecen, Debrecen, Hungary

**Keywords:** comorbidity, multimorbidity, rheumatic diseases, rheumatoid arthritis (RA), treat-to-target

The growing need to deliver more tailored and holistic patient care for people living with long-term rheumatic and musculoskeletal diseases (RMDs), has marked the onset of a new era in rheumatology. The shift away from the RMD itself as the “index” disease toward a focus on the patient living with RMD along with other comorbidities i.e., multimorbidity defined as the coexistence of two or more chronic diseases in the same individual (1), has placed emphasis on the importance of preventing, screening, and managing comorbidities in order to improve patient care. The concepts of multimorbidity and comorbidity both refer to a patient with more than one disease, but from a different perspective and are not mutually exclusive or contradictory (1). Whereas, comorbidity focuses on the index disease, addressing the co-occurrence of any distinct additional entities, in multimorbidity no index disease is defined and all morbidities are regarded of equal importance. An increasing body of evidence suggests that comorbidities jeopardize the achievement of treat-to-target goals and are a barrier to optimal treatment outcomes, an observation primarily originating from inflammatory arthritis studies including those in rheumatoid arthritis (RA) (2–5) and spondyloarthritides (SpA) (6, 7).

The appreciation of the importance in appropriately identifying and managing comorbidities in the routine care of people with RMDs, is the rationale for this themed issue. Through a series of articles that address key aspects relating to prevalence and screening of comorbidities in RMDs and impact on disease outcomes, this issue aims to inform the reader of the multi-faceted aspects of various comorbidities in RMDs. Methodological aspects relating to the assessment of comorbidities and their collective burden on disease outcomes are also discussed, aimed at informing the reader of available “tools” for recording and addressing comorbidities for clinical and research/epidemiological purposes.

Cardiovascular disease (CVD) in systemic rheumatic diseases represents an important comorbidity seen even in the absence of traditional cardiovascular (CV) risk factors and adding to the morbidity and mortality burden (8). Yet, CVD unfortunately remains poorly addressed in many rheumatology clinics. In the first article of this issue, Atzeni et al. discuss non-invasive and invasive techniques of investigating CVD including coronary artery disease (CAD), valve and other morphological and structural abnormalities. The techniques described range from trans-thoracic echocardiography to speckle Tracking Echocardiography (STE) to Positron Emission Tomography (PET) and three-dimensional ultrasound and demonstrate the spectrum of modalities currently available to assess CV morbidity. The article indicates the need for additional data before some of these modalities are applied in routine clinical practice, despite the improved diagnostic accuracy with some of the newer techniques.

Remaining on the topic of CVD in RMDs, Soulaidopoulos et al. discuss the role of statins in RA, highlighting the complex interplay between traditional risk factors (dyslipidaemia, insulin resistance, arterial hypertension, obesity, smoking), chronic inflammation and the development of premature atherosclerosis. The article discusses the potential beneficial impact of statins on RA disease activity, attributable to their anti-inflammatory and immunomodulatory properties, presenting the relevant literature. At the same time, the article discusses that while the use of statins is widely advocated in view of their crucial role in primary and secondary CVD prevention strategies, data on the precise benefit of such therapy in patients with RA are limited. The influence on lipid metabolism through systemic inflammation and anti-inflammatory treatments leads to variable states of dyslipidaemia in RA and results in differing indications for statin use across RA patients (9).

Rodríguez-Carrio et al. take a further leap into the realm of dyslipidaemia in immune-driven diseases, characterized by either decreased HDL levels or impaired HDL functionality. Altered HDL levels and blood lipids have been described in systemic autoimmune diseases, and the authors refer that the “paradoxical” effect on CV risk (the so-called “lipid paradox,” whereby low lipid levels are associated with increased CV risk) is widely accepted albeit poorly understood (10). Through appraising a broad range of immune-driven conditions, the study confirms that anti-HDL antibodies are a common hallmark in autoimmunity, especially in systemic diseases such as mixed connective tissue disease (MCTD) or ANCA-associated vasculitis. In addition, anti-HDL antibodies interplay with disease-related autoantibodies to account for the impaired antioxidant activity of HDL in these conditions. The study adds to the growing literature base on lipoprotein dysfunction and raises the potential usefulness of anti-HDL antibodies as emerging biomarkers for the unmet clinical need of CV risk stratification in these conditions.

Risk stratification in RMDs represents an exciting new avenue, with major research studies focusing on the identification and performance of various biomarkers in disease. The presence of cyclic citrullinated peptide (CCP) antibody in RA is known to predict the development of disease up to a decade before its actual onset and is also an indicator of more aggressive disease (11). Twigg et al. attempt to compare comorbidities in a cohort of CCP positive individuals without or prior to onset of inflammatory arthritis using the IACON cohort [the Inflammatory Arthritis CONtinuum study (IACON)]. Interestingly, no significant difference in comorbidities was observed among people with CCP antibodies without inflammatory arthritis, compared to those of patients with established inflammatory arthritis. The results of this study indicate that comorbidities in inflammatory arthritis may begin to accumulate before the onset of clinically apparent inflammation. Such information is useful and emphasizes yet again the importance of early screening for comorbidities.

Moving into SpA, Moltó and Nikiphorou discuss common comorbidities based on multinational epidemiological data raising awareness on the subject, this time in SpA. The article highlights the need for bridging the gap between recommendations for managing comorbidities and actual implementation in clinical practice. Osteoporosis and CVD are among the commonest reported comorbidities in SpA, an observation that may in part represent the outcome of differing screening processes and reporting of comorbidities. Aslam and Khan touch on this very issue, discussing how the lack of a gold standard in measuring and recording comorbidity and its burden on disease outcomes has resulted in specific indices being developed in recent years for use in RMDs. Such examples include the Rheumatic Disease Comorbidity Index (RDCI) (12) and Multimorbidity Index (MMI) (13). These indices have been developed primarily for research purposes and this article reflects how the study of comorbidities is critical in research, also in informing clinical practice.

Finally, shifting into other systemic autoimmune diseases and comorbidities, Alunno et al. remind us of lymphoma and lymphomagenesis in primary Sjogren's syndrome (pSS), important extra-glandular manifestations of disease that worsen prognosis. Non-Hodgkin's lymphoma is one of the commonest reported comorbidities in patients with pSS. Over the last 40 years, a consistent number of studies have attempted to identify clinical, serological, and histological predictors of lymphoma in pSS patients, but although progress has been made in this field, many aspects remain to be fully elucidated. Several clinical, serological, and histopathological features have been proposed as predictive for lymphoma in pSS patients, allowing early diagnosis and consequently, better management and prognosis. This review article encompasses the body of evidence published so far in this field highlighting the challenges and pitfalls of different biomarkers within clinical practice. It also provides an overview of epidemiological data, diagnostic procedures, and evidence-based treatment strategies for NHL in pSS. The article highlights that in clinical practice, clinicians should be alert to patients displaying clinical and serological features such as low C4 and persistent parotid gland enlargement. On the other hand, the possible association between histological evidence of germinal centers in salivary gland tissue and lymphoma development remains to be defined.

In conclusion, we hope that this themed issue increases awareness into comorbidities in RMDs and their importance, both from a clinical, but also epidemiological perspective. Comorbidities add to the burden of disease and should form a core part of the management of people with RMDs in routine clinical care, with emphasis placed on early recognition and management.

## Author contributions

EN wrote the first draft of the Editorial, which was reviewed by MN and ZS before producing a further draft. The final manuscript was reviewed and approved by all co-authors.

### Conflict of interest statement

The authors declare that the research was conducted in the absence of any commercial or financial relationships that could be construed as a potential conflict of interest.
